# Molecular Encapsulation of Histamine H_2_-Receptor Antagonists by Cucurbit[7]Uril: An Experimental and Computational Study

**DOI:** 10.3390/molecules21091178

**Published:** 2016-09-06

**Authors:** Hang Yin, Runmiao Wang, Jianbo Wan, Ying Zheng, Defang Ouyang, Ruibing Wang

**Affiliations:** State Key Laboratory of Quality Research in Chinese Medicine, Institute of Chinese Medical Sciences, University of Macau, Taipa, Macau 999078, China; hyin531@outlook.com (H.Y.); kamileowang@gmail.com (R.W.); jbwan@umac.mo (J.W.) yzheng@umac.mo (Y.Z.); defangouyang@umac.mo (D.O.)

**Keywords:** cucurbit[7]uril, H_2_-receptor antagonists, molecular modelling, complexation, host-guest interaction

## Abstract

The histamine H_2_-receptor antagonists cimetidine, famotidine and nizatidine are individually encapsulated by macrocyclic cucurbit[7]uril (CB[7]), with binding affinities of 6.57 (±0.19) × 10^3^ M^−1^, 1.30 (±0.27) × 10^4^ M^−1^ and 1.05 (±0.33) × 10^5^ M^−1^, respectively. These 1:1 host-guest inclusion complexes have been experimentally examined by ^1^H-NMR, UV-visible spectroscopic titrations (including Job plots), electrospray ionization mass spectrometry (ESI-MS), and isothermal titration calorimetry (ITC), as well as theoretically by molecular dynamics (MD) computation. This study may provide important insights on the supramolecular formulation of H_2_-receptor antagonist drugs for potentially enhanced stability and controlled release based on different binding strengths of these host-guest complexes.

## 1. Introduction

**Figure 1:**
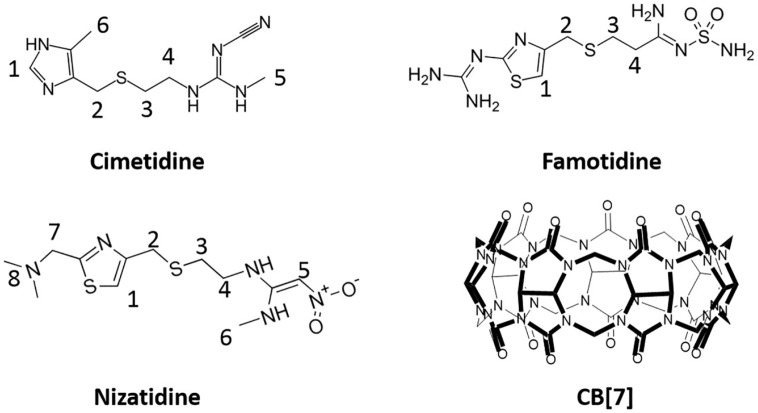
Molecular structures of cimetidine (CT), famotidine (FT), nizatidine (NT) and CB[7].

Among various macrocyclic molecules, an emerging family of molecular capsules known as cucurbit[*n*]urils (CB[*n*]s, *n* = 5–8, 10, 14) have recently attracted increasing attention in the field of pharmaceutical sciences and biomedical research [1,2]. CB[*n*]s consist of *n* glycoluril units that are connected by *n* pairs of methylene groups, with a lipophilic cavity in the middle that is accessible by a variety of guest molecules via two polar carbonyl-laced portals [3,4]. Due to its superior water-solubility, CB[7] (Figure 1) is a particularly attractive capsule to host a variety of guest molecules of biomedical and medical interest, both in vitro and in vivo [1,2].

On the one hand, CB[7]’s safety profile and biocompatibility has been well studied with several in vitro, in vivo and ex vivo models [5,6,7]. For instance, several in vitro studies on cell cultures have shown that CB[7] exhibits very low toxicity at up to 1 mM concentrations. The effects observed for an intravenous single dose *i.v.* injection with a mouse model demonstrated that CB[7] has a very low acute toxicity at a dose of 250 mg/kg, based on a body weight change of less than 10% within 5 days of the injections [5]. The tissue specific toxicity including neuro-, myo- and cardiotoxicity of CB[7] has been examined with the use of ex vivo electrophysiological models. The study reported that 1 mM of CB[7] did not exhibit statistically measurable neurotoxicity, although myotoxic and cardiotoxic activities were observed in the presence of CB[7] concentrations of 0.3 mM [6]. Very recently, we have studied the developmental and organ-specific toxicity profiles of CB[7] with live zebrafish models and concluded that CB[7] is relatively safe and biocompatible at functional levels, which is consistent with previous in vitro, ex vivo and in vivo results [7].

On the other hand, even before these preliminary investigations of CB[7]’s safety profile had been performed, over a decade ago the Collins [8] and Kim [9] research groups independently pioneered the use of CB[7] for encapsulation of platinum complex-based anti-cancer agents, and they have demonstrated that the toxicity of these agents was reduced upon molecular encapsulation by CB[7], presumably due to steric protection provided by the molecular capsule [8,9]. Following these works, CB[7] has drawn increasing attention as a host for the encapsulation of drug molecules in recent years. Along this line, our research group has investigated CB[7]’s encapsulations of a variety of biomedically important molecules during the past years, including imidazolium- and thiazolium-based model drugs [10,11], the photosensitizer norharmane [12], the anti-peptic ulcer drug ranitidine [13], the anticoagulant coumarin and the associated model drug coumarin-6 [14], vitamin B_12_ and its coenzyme [15], vitamin B_1_ [16], vitamin B_6_ [17], the neurotoxin MPTP/MPP^+^ [18], anesthetic agents such as tricaine [19], benzocaine and its metabolite *para*-aminobenzoic acid [20], as well as the anti-cancer drug camptothecin [21] and the anti-tuberculosis drug clofazimine [22]. It has been generally demonstrated by us and other researchers that drugs encapsulated within CB[7] may lead to one or more of several benefits including improved solubility, chemical stability, and therapeutic efficacy as well as reduced side-effects [14,18,19,21,22,23,24]. For instance, a molecular capsule of coumarin-6@CB[7] was shown to have significantly increased bio-uptake both in vitro and in vivo, in comparison with the free coumarin-6 [14]. Of biomedical relevance, we have demonstrated that CB[7] encapsulation of a neurotoxin MPTP in vivo may lessen the neurotoxicity of the guest molecule [18]. Additionally, we observed that encapsulation of anti-cancer drug camptothecin and anti-tuberculosis drug clofazimine by CB[7] has reduced these drug’s inherent toxicities and maintained their therapeutic efficacy, as demonstrated by both in vitro and in vivo evidence [21,22].

Similarly, the encapsulation of an anti-peptic ulcer drug ranitidine, which is also a histamine H_2_-receptor antagonist, by CB[7], protected the drug from thermal degradation, and this might be employed to extend the shelf-life of this drug and potentially enhance its therapeutic efficacy [13]. We have recently extended our study to several other histamine H_2_-receptor antagonists (Figure 1), namely cimetidine (CT), famotidine (FT) and nizatidine (NT), for their molecular encapsulation by CB[7] experimentally and computationally, and both sets of data have supported the formation of relatively strong 1:1 host-guest inclusion complexes between each of these drugs and CB[7], suggesting that computational methods may be used to predict drug-carrier interactions and preliminary drug formulation screening. More importantly, different binding affinities between each of these drugs with CB[7] may find application for potentially controlled release of this group of drug molecules.

## 2. Results and Discussion

### 2.1. Experimental Study of Molecular Encapsulations of Histamine H_2_-Receptor Antagonists

#### 2.1.1. ^1^H-NMR Studies of the Encapsulation Sites

**Figure 2:**
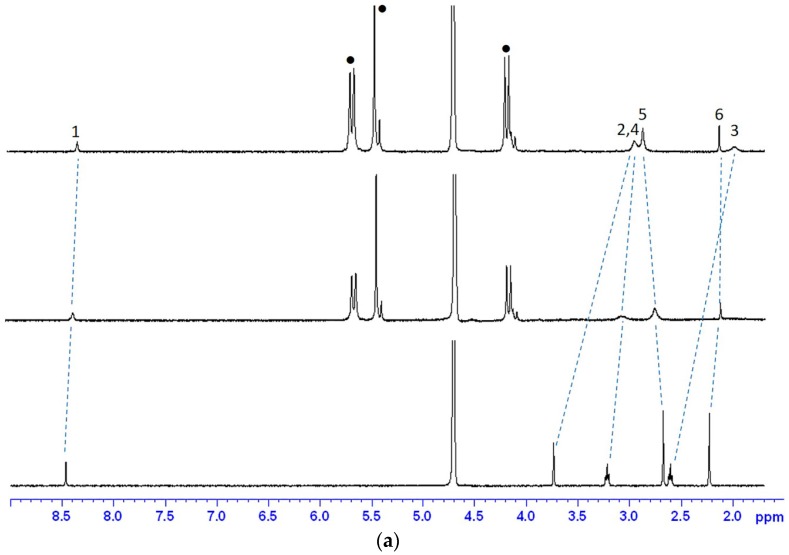

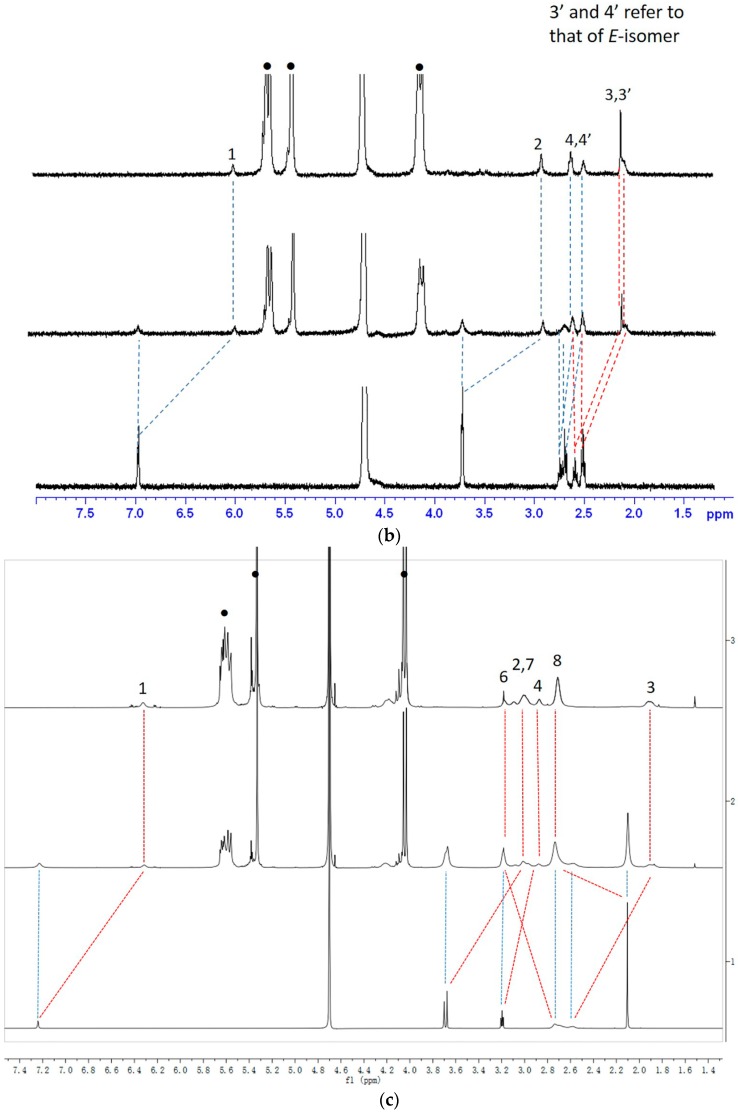
^1^H-NMR (400 MHz) spectra of (**a**) cimetidine; (**b**) famotidine and (**c**) nizatidine, in the absence and in the presence of 0.6 and 1.8 equiv. of CB[7] (with increasing CB[7] concentrations from bottom upwards) in D_2_O.

The binding sites of these molecular encapsulation complexes were examined by ^1^H-NMR spectroscopy. Generally, a proton resonance will shift to an upfield resonance if it is encapsulated within the cavity of CB[*n*], whereas it will shift to a downfield resonance if it is located outside of the cavity but close to the portal of the CB[*n*]. Protons that are well outside of the macrocyclic capsules would not exhibit any resonance shifts. As illustrated in Figure 2a, in D_2_O solution, the aromatic proton H(1), methyl protons H(6) and methylene protons (H(2), H(3) and H(4) protons) of CT have shifted upfield in the presence of CB[7] while only the methyl proton H(5) has shifted downfield, indicating that the entire aromatic ring and methylene groups are located within the cavity of CB[7] whereas the methyl on nitrogen is sitting outside of the cavity but near the portal. When insufficient CB[7] was present, appearance of only one set of NMR proton resonances of CT suggest that the exchange rate between the bound and free forms is fast with respect to the NMR time-scale.

Similarly, for FT, as shown in Figure 2b, the aromatic proton H(1) and methylene protons (H(2), H(3) and H(4) protons) of FT have shifted upfield in the presence of CB[7], suggesting that all these groups of FT are encapsulated within the cavity of CB[7]. In contrast with the case of CT, two separate sets of resonances when insufficient CB[7] was added into FT indicate that the exchange rate between the bound and free forms of FT is slow on NMR time-scale. For NT, as shown by Figure 2c, the aromatic proton H(1) and methylene protons (H(2), H(3), H(4) and H(7)) of NT have shifted upfield in the presence of CB[7] whereas the H(6) and H(8) protons have shifted downfield, corresponding to the encapsulation of these groups within the cavity of CB[7] and suggesting that the three nitrogen methyl groups are outside of the cavity. Because of the activation of the H(5) proton in D_2_O solution, the proton readily underwent exchange with deuterium, so that there was no visible signal in the NMR spectra. Like NMR spectra of FT, the two separate sets of resonances with insufficient CB[7] exhibited slow exchange rates between the bound and free forms of NT on the NMR time-scale.

#### 2.1.2. Job Plot and ESI-MS Studies

**Figure 3:**
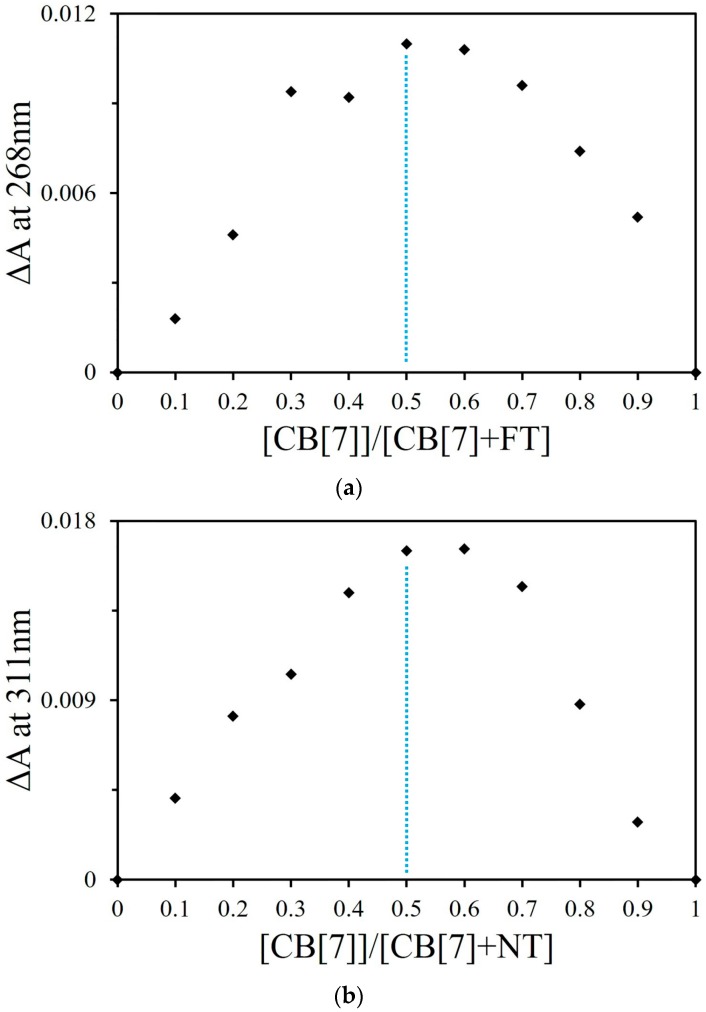
Job plots of FT@CB[7] complexes (**a**) and NT@CB[7] complexes (**b**).

The binding stoichiometries of FT-CB[7] and NT-CB[7] were studied by the continuous variation titration (Job plot) and monitored by UV-visible spectroscopy in deionized water (Figure S1). During the Job’s method titration, the total concentration of the guest and the host were the same in each sample. If a Job plot shows a maximum at 0.5 (the ratio of the concentration of the host to the total concentration of the host and the guest), it suggests that the binding stoichiometry between the host and the guest is 1:1. The Job plot of FT@CB[7] monitored by UV-visible spectroscopy at 269 nm (Figure 3a) reached a maximum at the ratio of 0.5 for (CB[7])/([CB[7]] + [FT]), suggesting that the binding ratio of FT and CB[7] is mainly 1:1. Similarly for NT, the Job plot of NT@CB[7] (Figure 3b) also reached a maximum at 0.5, implying that the binding ratio of NT and CB[7] is mainly 1:1 at this concentration level used in this study. Because the absorbance of CT monitored by UV-visible spectroscopy was not influenced by the addition of CB[7], the binding stoichiometry of CT@CB[7] could not be examined by this method. Additionally, the binding stoichiometries of all three complexes were also examined by ESI-MS analysis, which may provide direct evidence of the binding ratio of these supramolecular complexes. As expected (Figure S2), a doubly charged *m/z* peak (*m/z* = 708.23, calculated at 708.24) for CT-CB[7] sample indicated a 1:1 binding stoichiometry for this complex. Similarly, the doubly charged *m*/*z* peaks for the FT@CB[7] complex (*m*/*z* = 750.70, calculated at 750.70) and NT@CB[7] complex (*m*/*z* = 747.73, calculated at 747.74) supported that of both these two complexes existed as 1:1 host-guest pairs.

#### 2.1.3. Binding Affinity Studies by UV-Visible and NMR Spectroscopic Titration

**Figure 4:**
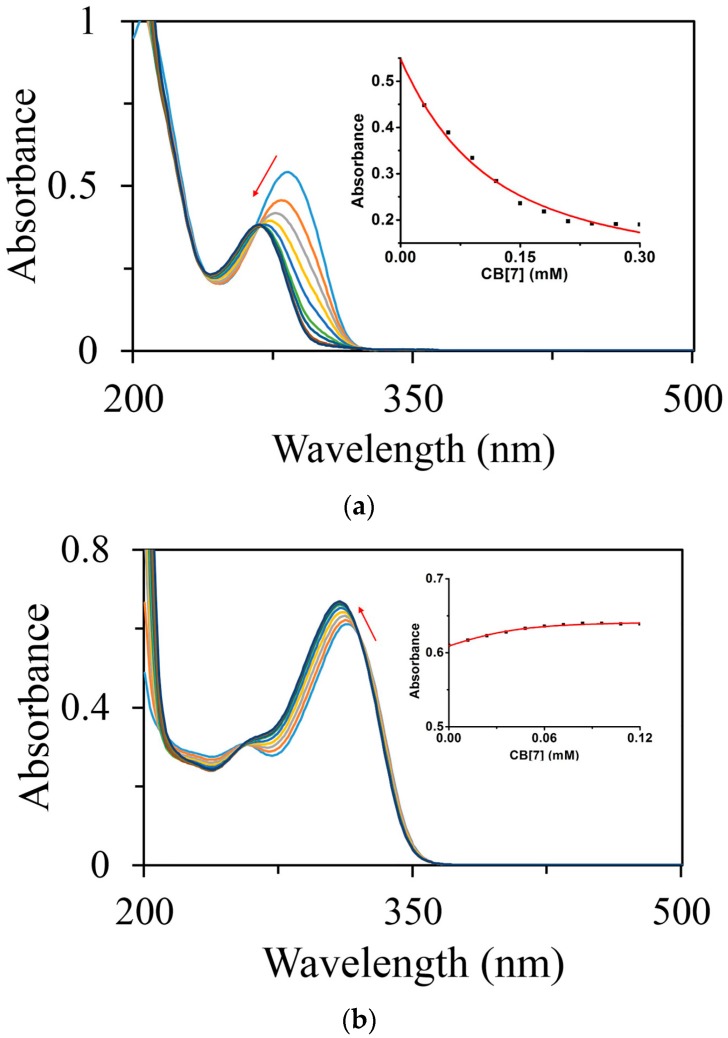

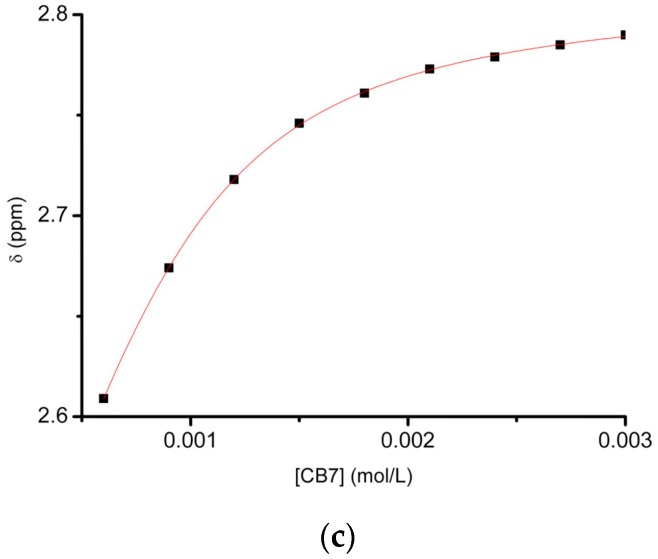
Absorbance spectra along with the corresponding fitting curves of FT@CB[7] (**a**) and NT@CB[7] (**b**) as monitored by UV-visible spectrometry. Fitting curve of H(5) proton NMR resonance of CT against the concentration of CB[7] in D_2_O at 25 °C (**c**).

Binding affinity is always considered as a key parameter to evaluate non-covalent binding behavior in host-guest interactions. With gradual addition of increasing amounts of CB[7] from 0 to 6.0 equivalents to a solution of 0.05 mM FT in deionized water, the absorbance at 283 nm monitored by UV-visible spectroscopy, gradually decreased (Figure 4a). A modest hypsochromic shift was observed, presumably due to the aromatic ring of FT was encapsulated in the cavity of CB[7]. The fitting curve of the absorbance at 283 nm against the host concentration showed good agreement with a 1:1 binding stoichiometry model and provided a binding constant *K*_a_ = 1.30 (±0.27) × 10^4^ M^−1^. The binding affinity of NT@CB[7] was examined by the same method as that used for FT@CB[7]. With the gradual addition of increasing amounts of CB[7] from 0 to 3.0 equivalents to a solution of 0.04 mM NT, the absorbance at 315 nm increased with a modest hypsochromic shift (Figure 4b). The fitting curve of the absorbance at 283 nm against the concentration provided a binding constant *K*_a_ = 1.05 (±0.33) × 10^5^ M^−1^ and also gave evidence to support a 1:1 binding stoichiometry.

Due to the lack of responsiveness of the CT’s absorbance to complexation with CB[7], the binding behaviors of CT@CB[7] was instead monitored by ^1^H-NMR spectroscopy in D_2_O. During the ^1^H-NMR titration, the concentration of CT was kept constant at 1.0 mM in the presence of an increasing concentration of CB[7] (from 0 to 3.0 equivalents). The change in the chemical resonances was clearly observed (Figure S3), and the H(5) proton of CT was chosen as a probe for the binding behavior because the resonance of H(5) proton was the most trackable one among all of the protons. The non-linear least squares fitting curve (Figure 4c) of the chemical resonance of H(5) against the concentration of CB[7] yielded a binding affinity of *K*_a_ = 6.57 (±0.19) × 10^3^ M^−1^.

#### 2.1.4. ITC Titration Studies

Isothermal titration calorimetry (ITC) is a powerful technique to give not only binding stoichiometry (N) and binding affinity (*K*_a_) but also thermodynamic parameters (∆*H*, ∆*G* and T∆*S*) by monitoring the micro-level thermal change of a given system. An aqueous solution of CB[7] for ITC was loaded into the titration syringe and solutions of CT, FT, or NT in deionized water were loaded in the titration cell, individually, and ITC titration analysis of these samples was conducted at 25 °C. The binding parameters including N (binding stoichiometry), *K*_a_ (binding affinity), ∆*H* (enthalpy change), ∆*G* (Gibbs free energy change) and T∆*S* (entropy change), was auto-analyzed by MicroCal PEAQ-ITC Analysis Software 1.1.0.1262 (for ITC titration isotherms, refer to Figure S4), and summarized in the Table 1.

ITC tests have further confirmed the 1:1 binding stoichiometry of these complexes, consistent with Job plots and ESI-MS results. For all of the three complexes, enthalpy change was the main contributor to these non-covalent interactions, suggesting hydrogen bondings and ion-dipole interactions are the main driving forces. This is likely due to strong interactions between protonated amines of the drug molecules and carbonyl portals of the host. Of note, the binding affinities derived from ITC tests are different from, but generally comparable with, the previously discussed results from UV-visible and NMR spectroscopic titrations. The most significant difference between *K*_a_ values derived from ITC and NMR/UV-Vis methods is for the NT@CB[7] complexes, where the *K*_a_ from UV-Vis titration is approximately 7.8 fold of the value from ITC titration. To the best of our knowledge, the much difference in *K*_a_ values is reasonable as binding constants may vary quite significantly if different methods are used [25]. Additionally, the binding constants varying from 10^3^ to 10^5^ in aqueous solutions seem to be comparable with those recently observed on other drug-CB[7] complexes [14,16,20,22], attesting the potential of these complexations in biomedical sciences.

According to the formula: Δ*G* = −*RT*ln*K* (*T* = 297.5 K), the Δ*G* values of the complexes could be readily calculated from the experimental results based on *K*a value derived from the UV-visible and NMR spectroscopic titrations (Δ*G*_CT_ = −5.21 kCal/mol, Δ*G*_FT_ = −5.62 kCal/mol and Δ*G*_NT_ = −6.85 kCal/mol). These values are generally consistent with the Gibbs free energy changes measured by ITC methods (Table 1).

### 2.2. Computational Study of Host-Guest Complexes

**Figure 5:**
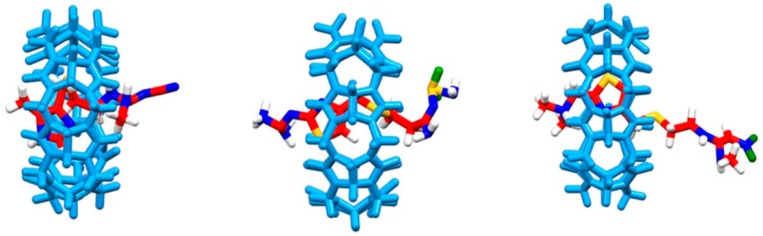
Snapshots of CT@CB[7] (**left**), FT@CB[7] (**middle**) and NT@CB[7] (**right**) at 30 ns.

CT, FT and NT are generally hydrophilic molecules with hydrophobic moieties, while CB[7] has a hydrophobic cavity. Therefore, the water molecules have stronger influence on the guest molecules and their binding with CB[7] in these systems. Figure 5 showed the complexes’ structures of these drug@CB[7] complexes by molecular dynamics (MD) simulation where water molecules can be built in. The aromatic ring and methylene groups of CT, FT and NT are located within the cavity of CB[7], which are consistent with the binding geometries derived from our experimental results (Figure 2), as our NMR experiments showed that the entire aromatic ring and methylene groups of CT were located within the cavity of CB[7] whereas the methyl on nitrogen is sitting outside of the cavity but near the portal. The aromatic and methylene groups of FT and NT were encapsulated within the cavity of CB. Three nitrogen methyl groups of NT were outside of the cavity.

Table 2 shows the thermodynamic parameters including enthalpy and entropy changes as well as Gibbs free energies of the complexes that were calculated by the MM_GBSA method [26]. Interestingly, the calculated data also suggested that enthalpy changes are the main contributors towards these non-covalent interactions whereas entropy changes contributed negatively, consistent with our ITC results (Table 1). Entropic contribution is associated with the changes of water molecules within the CB[*n*] cavity, but this might be compensated by the ordered structure of host-guest self-assembly and associated water molecules by the portals of the host molecules. The observed discrepancy in values between the calculated and the experimental methods has been observed frequently before, as different experimental and modeling approaches may have result in different values. There are two possible reasons for such differences: on the one hand, the systems of MD simulation were ideal systems with only one ligand, one macrocycle and water molecules, while the real solutions contained much more solute and solvent molecules; on the other hand, the force field may be necessary to be further optimized for CB[7] systems, which deserves further in-depth investigations.

## 3. Materials and Methods

### 3.1. Materials

CB[7] was synthesized according to a literature method [27,28]. CT, FT and NT were purchased from TCI^®^ (Shanghai, China) and used as received.

### 3.2. Instrumentation

The ^1^H-NMR spectra were acquired using an Ultra Shield 400 PLUS NMR spectrometer (Bruker, Karlsruhe, Germany). The ESI-MS spectrometry analysis was conducted using a LTQ OrbiTrap XL instrument (Thermo, Waltham, MA, USA) equipped with an ESI/APcI multiprobe. The UV-visible spectroscopic analysis was performed using a DR6000 UV-visible spectrometer (HACH, Düsseldorf, Germany) with a 1.0 cm path length quartz cell. The isothermal titration calorimetry data was studied with a PEAQ-ITC instrument (Malvern, Northampton, MA, USA).

### 3.3. Complexes Preparation and Characterization

Stock solutions of CT, FT and NT, each at 1 mM concentration, were prepared with Milli-Q water (Merck Millipore, Darmstadt, Germany). The solutions of FT and NT for the Job plot titration and binding affinity titration were prepared by diluting the stock solution to concentrations of 0.05 and 0.04 mM, respectively. The solutions of CB[7] for the Job plot titration were prepared by diluting 1 mM CB[7] to the same concentration as the solutions of FT and NT, respectively. The complexes solutions of FT and NT employed for the binding constant titrations were 0.05 mM FT in the presence of 6.0 equivalents of CB[7], and 0.04 mM NT in the presence of 3.0 equivalents of CB[7], respectively. Due to the poor optical properties of CT and the fact that CB[7] encapsulation doesn’t influence its absorbance properties, both the Job plot titration and binding constant titration of CT@CB[7] were determined by ^1^H-NMR analysis. The solutions of CT for the binding titration by ^1^H-NMR analysis were prepared by dissolving 1 mM CT in D_2_O in the absence and in the presence of various amounts of CB[7] (up to 3.0 equivalents). The ITC solutions of 0.05 mM CT, 0.05 mM FT and 0.1 mM NT were diluted from their 1 mM stock solutions, respectively. The ITC solution of CB[7] was prepared at 2 mM with Milli-Q water.

### 3.4. MD Computation

MD simulations were performed using the Amber14 and Amber Tools 14 software package [29]. All molecules were built using the Leap module with Amber GAFF force field and Antechamber module by AM1-BCC charge method. The Amber Tools 14 were used to build the starting structure of the drug and CB[7], as well as drug-CB[7]complexes with TIP3P water model of 10 Å (Table 3). After energy minimization, 30-ns simulations were performed and the protocol was similar to those described in our previous publications [30,31,32]. The MM_GBSA method was used to calculate the enthalpy (Δ*H*) and the Normal Mode Analysis was performed for the entropy (TΔ*S*) [26]. The 100 MD snapshots from the last 1 ns of each system were used for binding free energy calculations.

## 4. Conclusions

In summary, we have investigated both experimentally and computationally the molecular interactions between a promising drug carrier cucurbit[7]uril and a group of H_2_-histamine antagonists, including cimetidine, famotidine and nizatidine. We have found that the non-covalent interactions between these drugs and the host molecules are mainly enthalpy driven, as hydrogen bonding and ion-dipole interactions play a dominant role in the complexation processes. In addition, molecular modeling techniques could help us investigate the binding behaviors between these drugs and a macrocyclic carrier and in this case confirmed that these complexations are mainly enthalpy driven. These results, taken together, support the use of CB[7] as a potential drug carrier with tunable binding strength that may ultimately benefit formulation and delivery of specifically selected drugs.

## Figures and Tables

**Table 1:** Thermodynamic parameters of non-covalent interactions of CT@CB[7], FT@CB[7] and NT@CB[7] encapsulation complexes at 25 °C, derived from ITC, NMR and UV-vis titrations (all uncertainties are standard deviations).

Complexes	Method	N	*K*_a_ (M^−1^)	Δ*H* (kCal/mol)	Δ*G* (kCal/mol)	TΔ*S* (kCal/mol)
CT@CB[7]	ITC	0.99	1.44 (± 0.38) × 10^4^	−13.23 ± 4.01	−5.69 ± 0.16	−7.57 ± 0.01
	NMR		6.57 (± 0.19) × 10^3^		−5.21 ± 0.02	
FT@CB[7]	ITC	1.12	4.95 (± 0.36) × 10^4^	−11.08 ± 0.38	−6.40 ± 0.04	−4.68 ± 0.01
	UV-vis		1.30 (± 0.27) × 10^4^		−5.62 ± 0.12	
NT@CB[7]	ITC	0.95	1.34 (± 0.21) × 10^4^	−15.60 ± 2.18	−5.64 ± 0.09	−9.96 ± 0.01
	UV-vis		1.05 (± 0.33) × 10^5^		−6.85 ± 0.19	

**Table 2:** Binding free energies (kCal/mol) of drug@CB[7] complexes by MM_GBSA method.

kCal/mol	CT@CB[7]	FT@CB[7]	NT@CB[7]
ΔE_TOT_	−37.55 ± 5.24	−33.12 ± 3.93	−32.47 ± 3.36
TΔS_TOT_	−20.10 ± 1.09	−20.86 ± 1.09	−19.68 ± 1.63
ΔG	−17.45 ± 4.92	−12.25 ± 3.77	−12.79 ± 2.97

**Table 3:** Simulation settings of the systems.

Parameter	CT-CB[7]	FT-CB[7]	NT-CB[7]
Water shell (Å)	10	10	10
Atom number of CB7	126	126	126
Atom number of drug	34	37	44
Atom number of Cl-	1	1	1
Number of water	1413	1736	1365
Total atom number	4400	5374	4265

## Supplementary Materials

Supplementary materials can be accessed at: http://www.mdpi.com/1420-3049/21/9/1178/s1.
