# Supplementary material for: Molecular Encapsulation of Histamine H2-Receptor Antagonists by Cucurbit[7]Uril: An Experimental and Computational Study
Source: Molecules. 2016 Sep 6;21(9):1178. doi: 10.3390/molecules21091178 (PMC6274153; doi:10.3390/molecules21091178)
Supplement: Supplementary file 1 [file molecules-21-01178-s001.pdf]

Supplementary Materials: Molecular Encapsulations of Histamine H_2-Receptor Antagonists by Macrocyclic Cucurbit[7]uril: An Experimental and Computational Study

Hang Yin, Runmiao Wang, Jianbo Wan, Ying Zheng, Defang Ouyang and Ruibing Wang

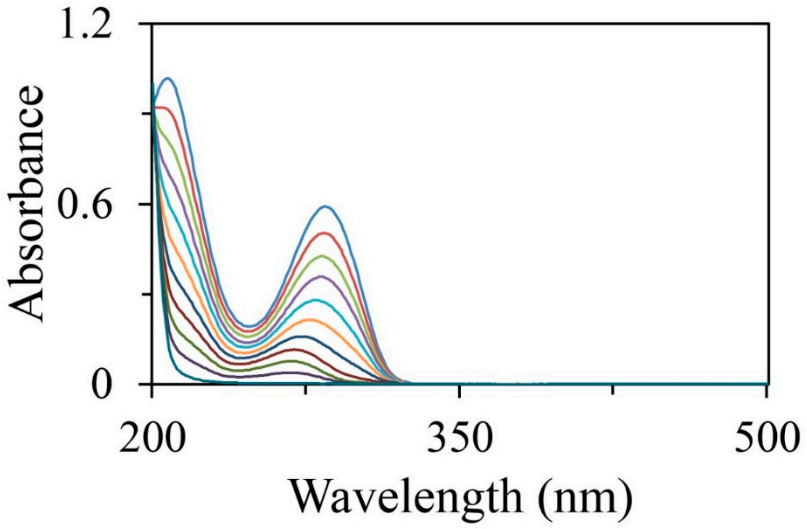

(**A**)

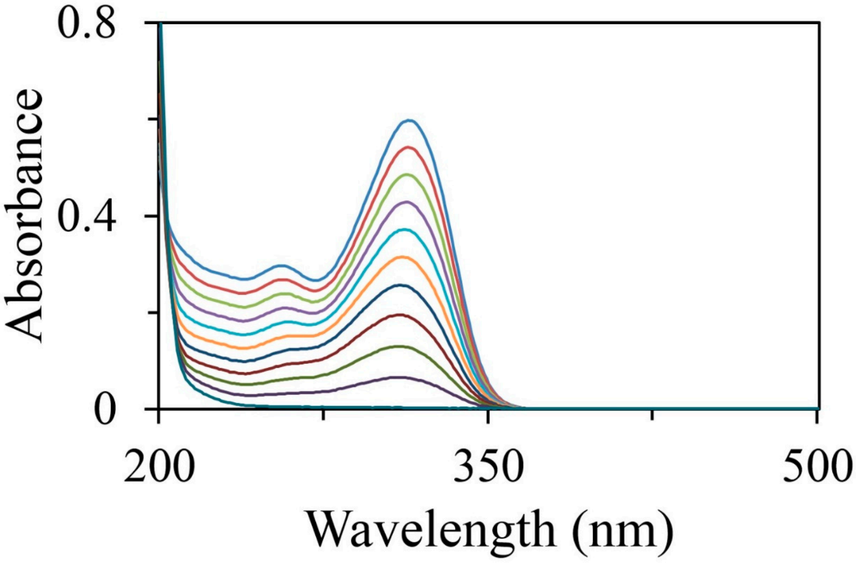

(**B**)

Figure S1. UV-Visible absorbance spectra of FT and CB[7] under Job plot titration (**A**); UV-Visible absorbance of NT and CB[7] under Job plot titration (**B**).

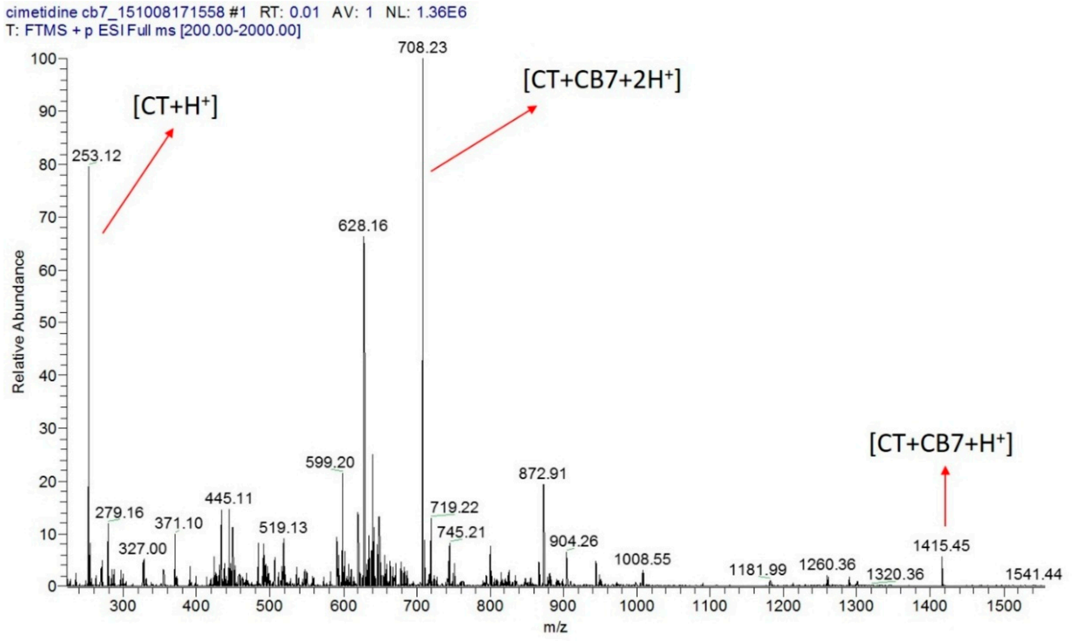
cimetidine cb7_151008171558 #1 RT: 0.01 AV: 1 NL: 1.36E6
T: FTMS + p ESI Full ms [200.00-2000.00]
Relative Abundance
m/z
[CT+H⁺]
[CT+CB7+2H⁺]
[CT+CB7+H⁺]
708.23
253.12
628.16
599.20
872.91
445.11
279.16
371.10
519.13
327.00
719.22
745.21
904.26
1008.55
1181.99
1260.36
1320.36
1415.45
1541.44

(**A**)

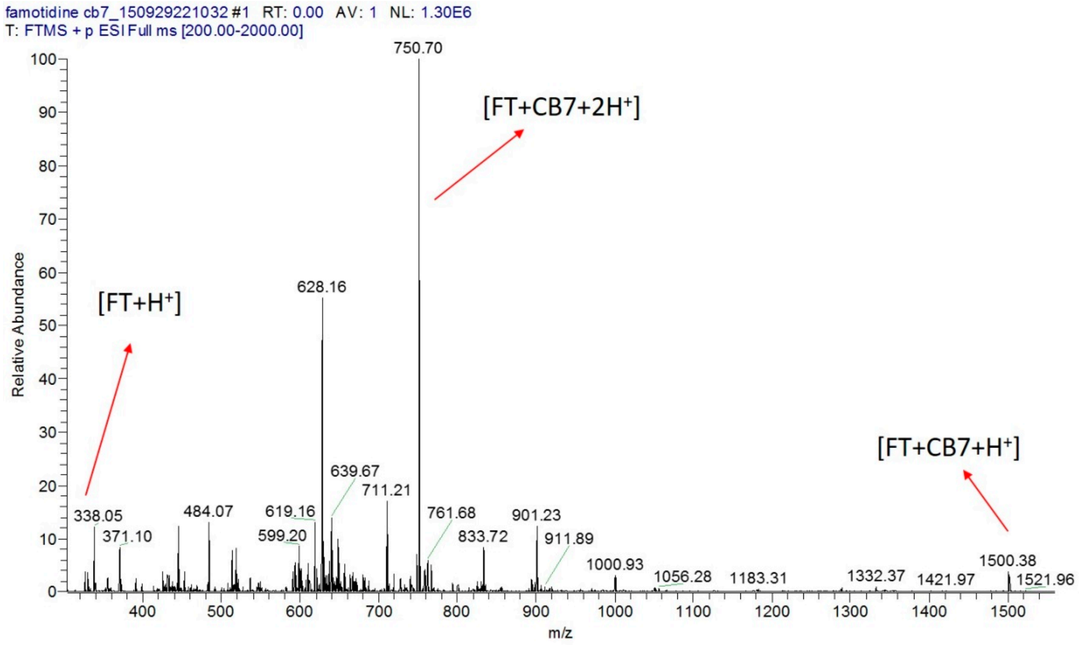
famotidine cb7_150929221032 #1 RT: 0.00 AV: 1 NL: 1.30E6
T: FTMS + p ESI Full ms [200.00-2000.00]
Relative Abundance
m/z
[FT+H⁺]
[FT+CB7+2H⁺]
[FT+CB7+H⁺]
750.70
628.16
639.67
711.21
338.05
371.10
484.07
619.16
599.20
761.68
833.72
901.23
911.89
1000.93
1056.28
1183.31
1332.37
1421.97
1500.38
1521.96

(**B**)

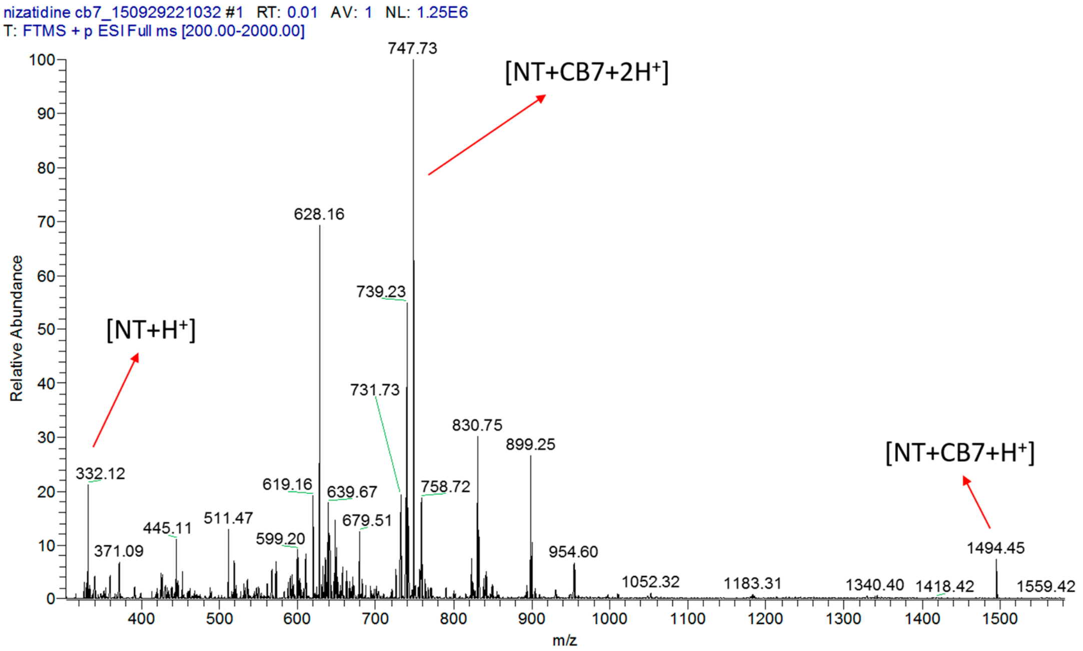

(**C**)

Figure S2. ESI-MS spectra of CT@CB[7] (**A**), FT@CB[7] (**B**) and NT@CB[7] (**C**).

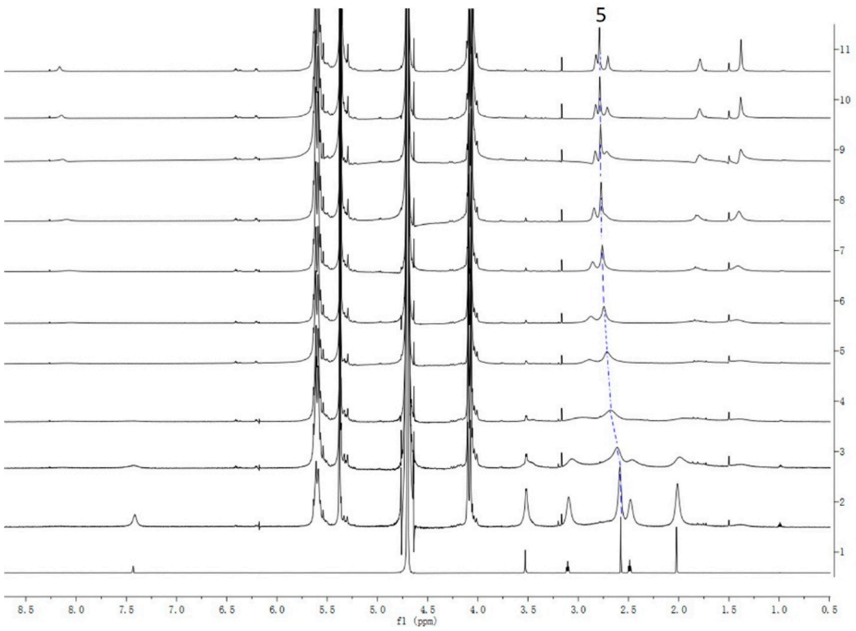

Figure S3. ^{1}H-NMR spectra of CT in the presence of increasing concentration of CB[7] (increased from 0 to 3.0 eqv. of CT).

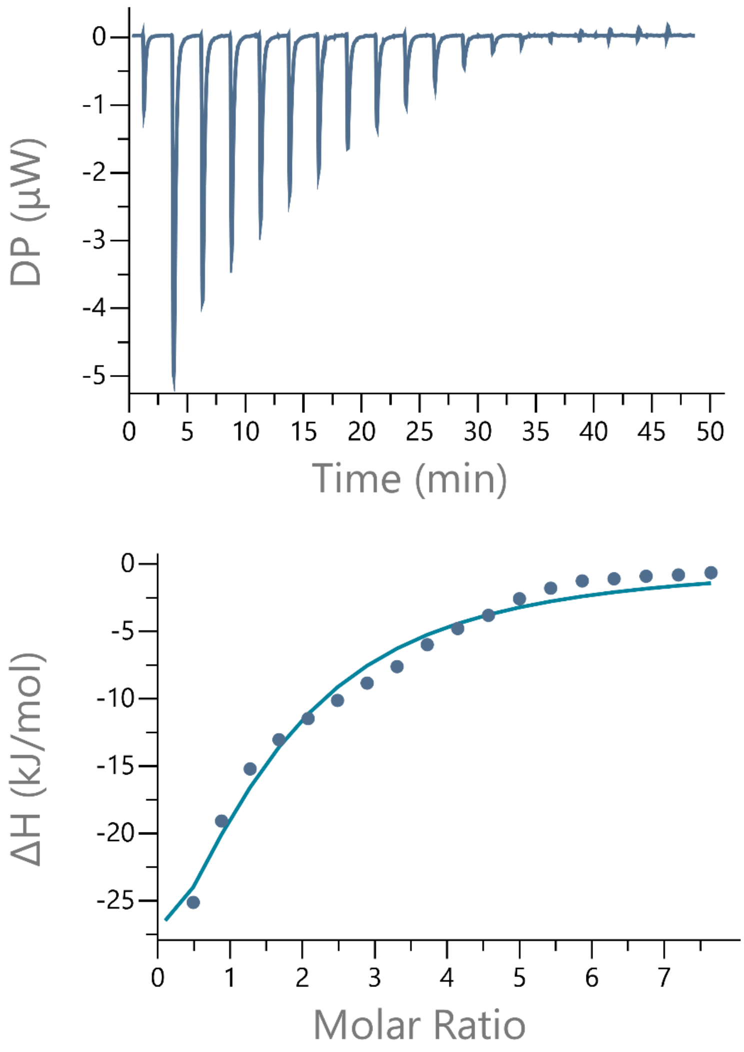
0
-1
-2
-3
-4
-5
DP (µW)
0
5
10
15
20
25
30
35
40
45
50
Time (min)
0
-5
-10
-15
-20
-25
ΔH (kJ/mol)
0
1
2
3
4
5
6
7
Molar Ratio

(**A**)

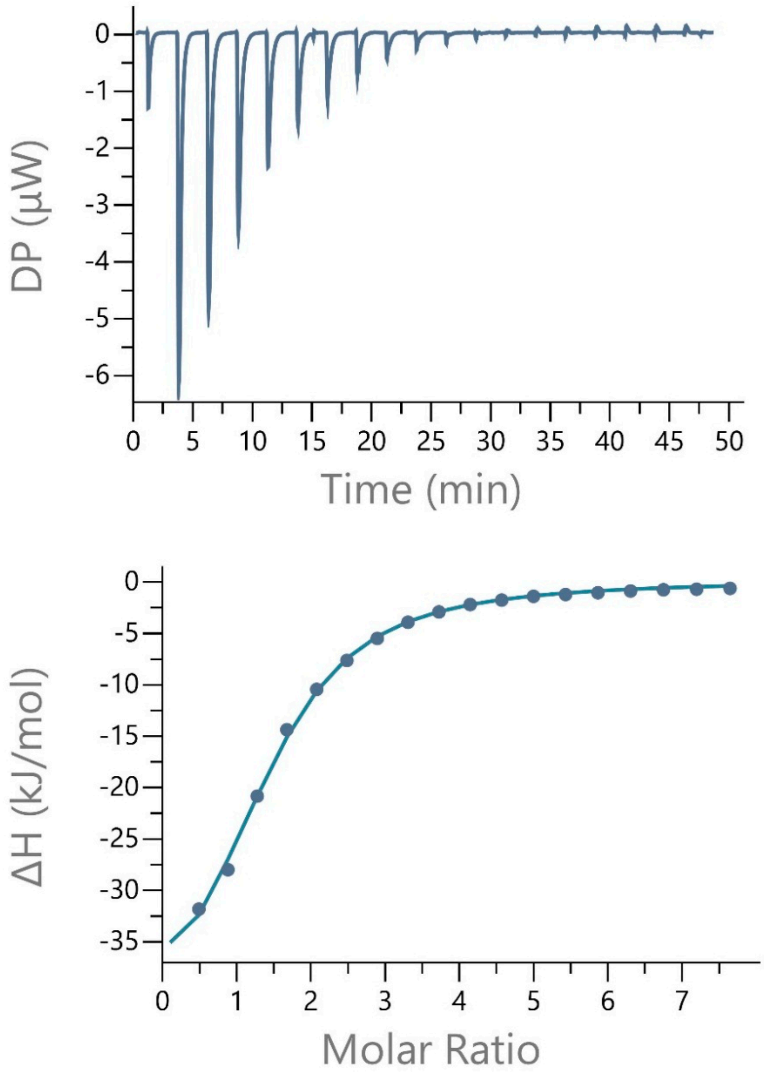

(**B**)

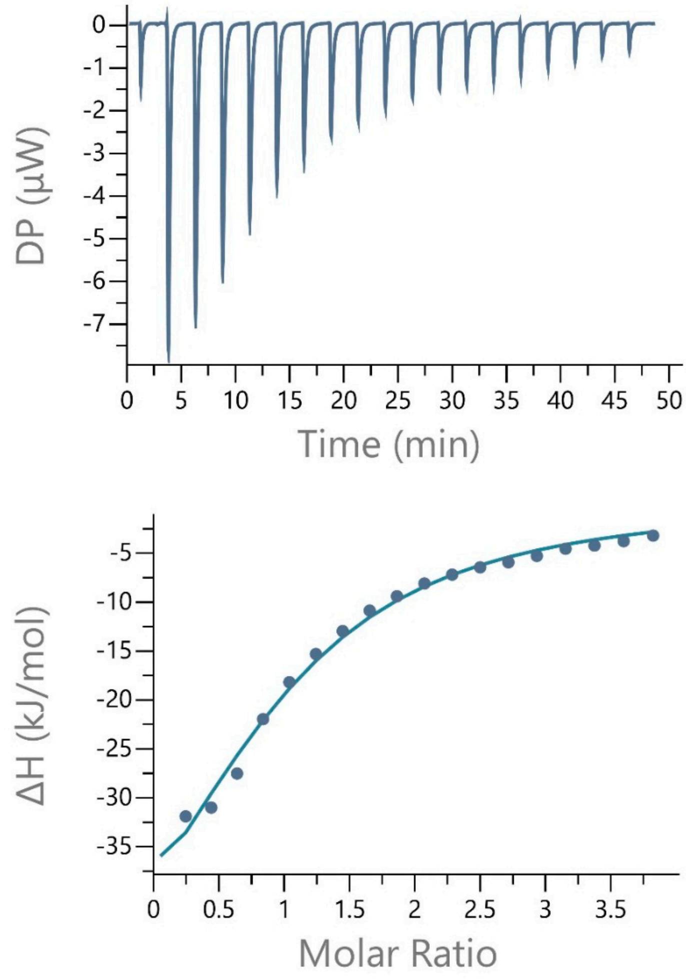

(**C**)

Figure S4. ITC raw plots and integrated heat plots of (**A**) CT with CB[7], (**B**) FT with CB[7], and (**C**) NT with CB[7].
